# Supplementary figures and images for: Borax-based gel electrophoresis: A novel approach for RNA integrity analysis
Source: PLoS One. 2026 Feb 27;21(2):e0344092. doi: 10.1371/journal.pone.0344092 (PMC12948047; doi:10.1371/journal.pone.0344092)

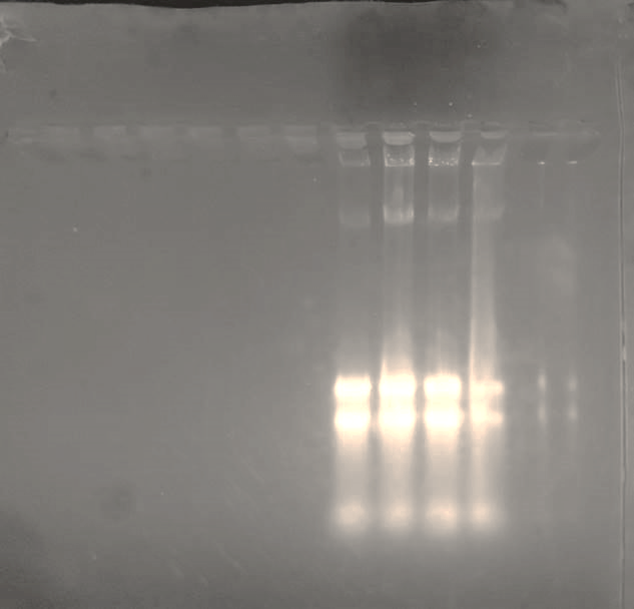

Supplement: S1 Fig — (TIF) [file pone.0344092.s001.tif]
