# Supplementary material for: Borax-based gel electrophoresis: A novel approach for RNA integrity analysis
Source: PLoS One. 2026 Feb 27;21(2):e0344092. doi: 10.1371/journal.pone.0344092 (PMC12948047; doi:10.1371/journal.pone.0344092)
Supplement: S1 File — Original uncropped and unadjusted blot/gel images. (PDF) [file pone.0344092.s002.pdf]

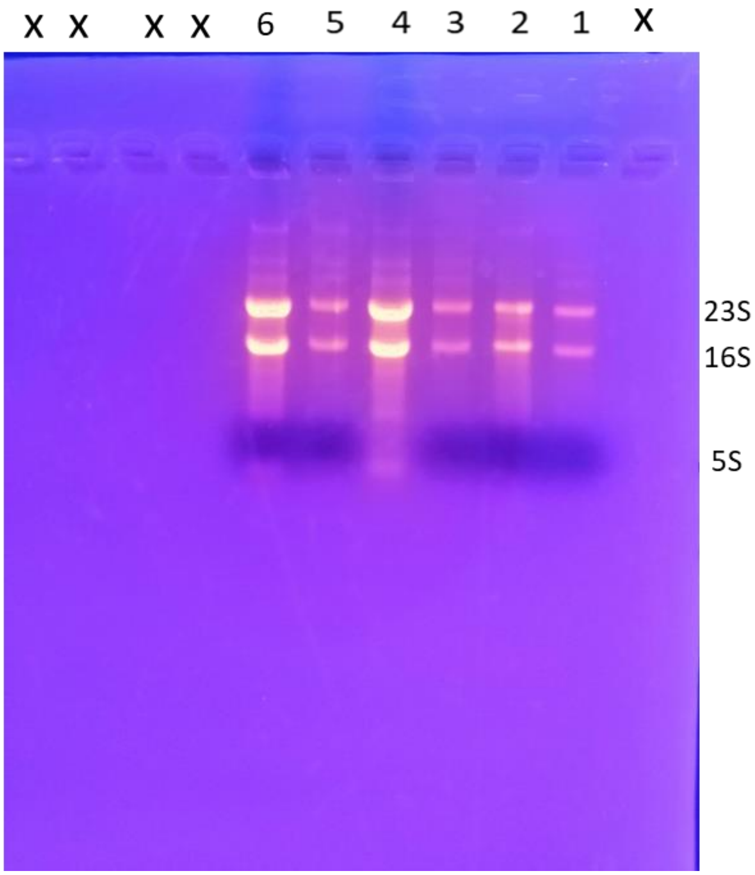

Fig 1: Electrophoretic Separation of *E. coli* Total RNA

Total *E. coli* RNA was separated by Borax Agarose Gel Electrophoresis to investigate the influence of various loading buffer formulations on migration patterns. The 0.8 % agarose gel was prepared with ethidium bromide (EtBr) integrated prior to casting. Electrophoresis was conducted at 120V for 25minutes. Lanes 1, 5, & 6: Total RNA loaded using a standard bromophenol blue loading buffer; lanes 2 & 3: Total RNA loaded using the standard buffer with Sodium Dodecyl Sulfate (SDS) omitted; lane 4: Total RNA loaded using a colorless buffer, with bromophenol blue omitted, x= empty lane.

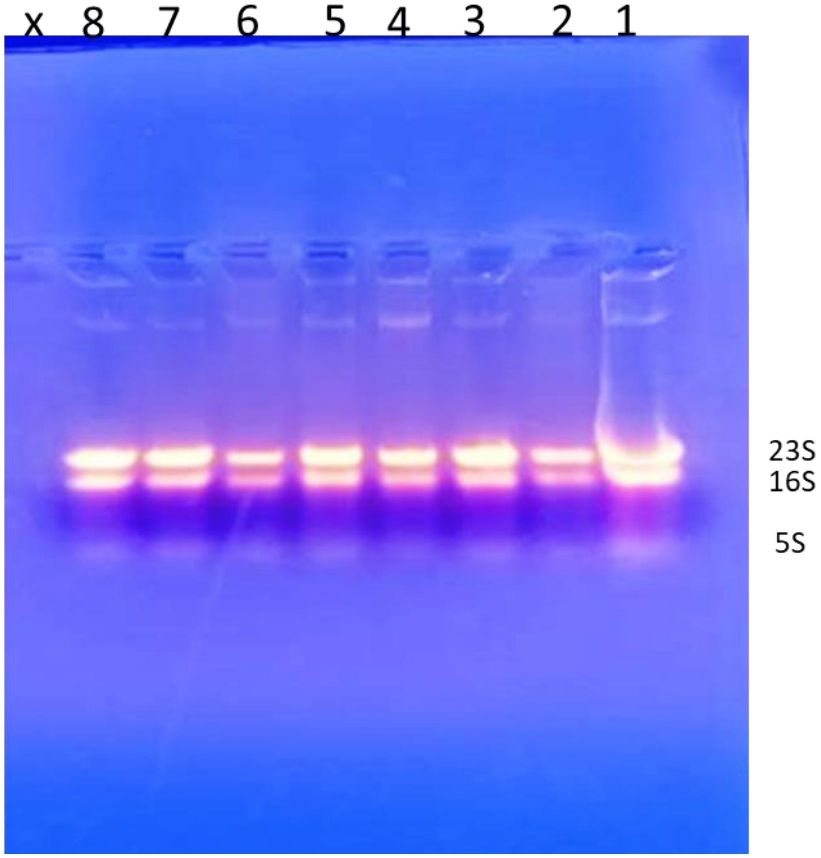

Fig 2. Effect of Low Borax Concentration Pre-treatment on Total *E. coli* RNA Integrity in TAE Agarose Gel.Total *E. coli* RNA samples were incubated with a range of low borax concentrations and subsequently separated using a standard TAE agarose gel electrophoresis system. Lane 1: RNA sample treated with 0 µM borax (Untreated Control). Lanes 2–7: RNA samples treated with borax concentrations of 50 µM, (Lane 2), 100 µM, (Lane 3) 150 µM, (Lane 4), 200 µM (Lane 5), 250 µM (Lane 6), and 300 µM (Lane 7), x= empty lane.

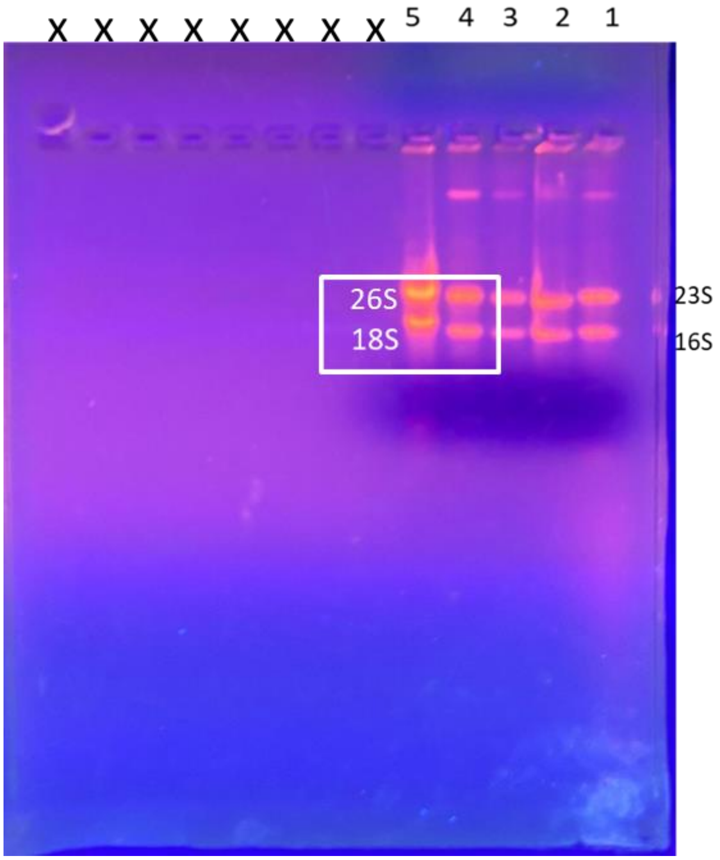

Fig 3. Universal Applicability of Borax-Based Gel Electrophoresis to Diverse Species
The figure demonstrates the successful separation and assessment of total RNA integrity extracted from diverse microbial species using the 5 mM borax agarose gel system. The method effectively resolves both prokaryotic ribosomal RNA (23S and 16S) and eukaryotic ribosomal RNA (26S/28S and 18S) species, confirming its utility across biological kingdoms. The RNA samples were loaded as follows:

- Lane 1: Total RNA from the Gram-negative bacterium *Pseudomonas aeruginosa*.
- Lane 2: Total RNA from the Gram-positive bacterium *Staphylococcus aureus*.
- Lane 3: Total RNA from the Gram-positive bacterium *Enterococcus faecalis*.
- Lane 4: Total RNA from the eukaryotic fungus *Candida glabrata*.
- Lane 5: Total RNA from the eukaryotic fungus *Candida albicans*
- X = empty lane.

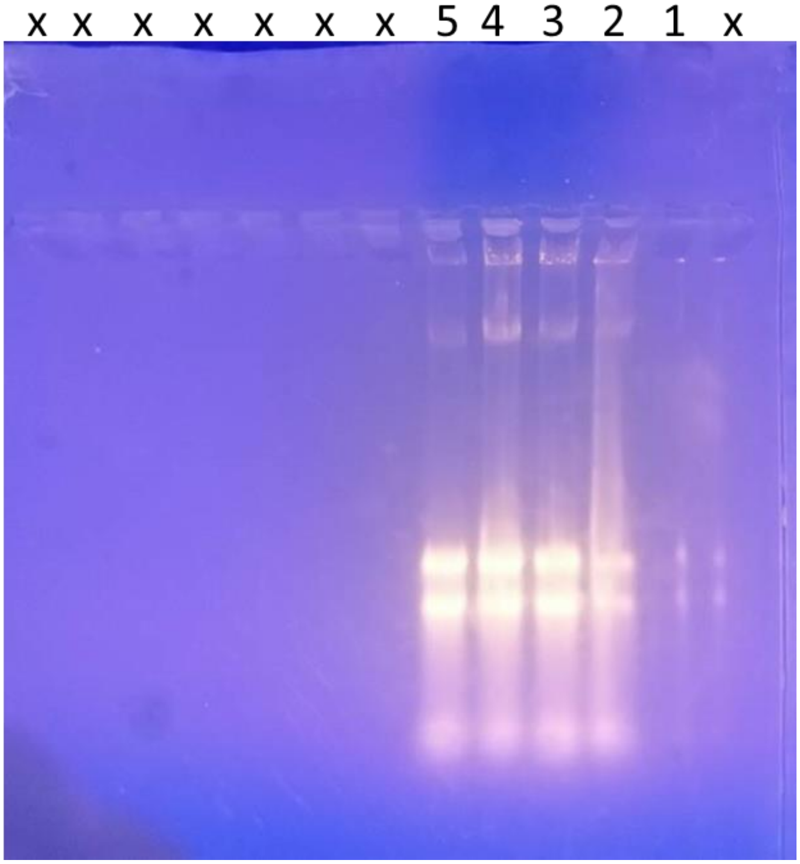

S1 Fig. Concentration dependent effects of borax on RNA integrity
