# Supplementary material for: Borax-based gel electrophoresis: A novel approach for RNA integrity analysis
Source: PLoS One. 2026 Feb 27;21(2):e0344092. doi: 10.1371/journal.pone.0344092 (PMC12948047; doi:10.1371/journal.pone.0344092)
Supplement: Attachment — Submitted filename: Rebuttal Letter.pdf [file pone.0344092.s004.pdf]

Rebuttal Letter (Response to Reviewers)

Title: Borax-Based Gel Electrophoresis: A Novel Approach for RNA Integrity Analysis **Manuscript Number:** PONE-D-25-34099 (Placeholder)

Dear Dr. Ho and Reviewers,

I thank you and the reviewers for the insightful and constructive comments on my manuscript. I appreciate the time taken for the careful review of my work and am pleased to submit a revised version that addresses all the concerns raised by the Academic Editor and the reviewers.

I believe the inclusion of new data on eukaryotic RNA and the detailed clarification of the loading buffer study significantly strengthen the paper, particularly its claim of universal applicability and simplified methodology.

Below is a detailed, point-by-point response to each comment. All changes I made have been highlighted in the manuscript titled **'Revised Manuscript with Track Changes.docx'**.

Academic Editor & Journal Requirements

**Req. #**	**Original Comment**	**My Response**
**AE #1**	Include other types of RNA, such as eukaryotic RNA, in vitro synthesized RNA or others in the analysis.	I agree this expansion was crucial for demonstrating the method's versatility. **I performed new experiments** and included data demonstrating the successful application of the borax method to total RNA extracted from **two eukaryotic fungal species** (*Candida glabrata* and *Candida albicans*), alongside the prokaryotic species. This new data confirms the borax buffer's capacity to resolve complex eukaryotic secondary structures (26S/28S and 18S bands). This information is presented in **Figure 3** and discussed in the Results section under "Universal Applicability."
**JR #2**	We note that you have included the phrase "data not shown" in your manuscript.	I have fully addressed this. I replaced the single instance of "data not shown" with a citation to a new Supporting Information file, which contains the relevant data: **(S1 fig)**. This file will be submitted separately.
**JR**	PLOS ONE now requires	I confirm that all original, uncropped, and unadjusted

Req. #	Original Comment	My Response
**#3**	that authors provide the original uncropped and unadjusted images underlying all blot or gel results.	raw images underlying the data in **Figures 1, 2, 3, and S1 Fig** have been compiled into a single file, which I will upload as Supporting Information titled **'Original Uncropped Images'** to meet this requirement.

Reviewer #1

Comment #	Original Comment	My Response
**1.**	The article shows little innovation, being more of a technical report than a paper focused on innovative science... I believe the findings could be substantially expanded through the use of different types of RNA (eukaryotic RNA, in vitro synthesized RNA, and circular RNAs).	I appreciate this feedback and have significantly expanded the scope and innovative value of the work. As noted above (AE #1), the study now includes data demonstrating the successful resolution of **eukaryotic RNA** (26S/28S and 18S bands) from two different *Candida* species (**Figure 3**). I argue that demonstrating the capacity of a non-toxic, non-formaldehyde method to resolve complex eukaryotic RNA secondary structures constitutes a significant innovation. Additionally, the revised manuscript includes detailed methods and results for my **loading buffer component experiment (Figure 1)**, which yielded a crucial practical discovery: that the absence of the BPB tracking dye significantly enhances the visualization of the 5S rRNA band, offering a clear optimization recommendation for users of this novel technique.

Reviewer #1

Comment #	Original Comment	My Response
**1.**	Add the method to obtain total RNA samples from *E. coli*.	**Done.** I have clarified the sample preparation protocol in the "Sample Preparation and RNA Extraction" section of the Methods to confirm that the identical acidic lysis protocol was applied to all species, including *E. coli*.
**2.**	In “Electrophoresis - Approximately 20 µL of RNA", what would be the RNA concentration in mg used?	**Done.** I have added the required concentration detail to the "Electrophoresis" section of the Methods, which now reads: "Approximately 20 µL **(containing approximately 1.5-2.0 µg of total RNA)** was mixed with..."
**3.**	In figure 1, add lane 1 and 2.	**Done.** I have fully revised the **Figure 1 Legend** to provide an explicit, lane-by-lane breakdown of all samples shown (Lanes 1-6), clearly differentiating between the Standard, SDS-Omitted, and **Colorless (BPB -Omitted)** loading buffer formulations used.
**4.**	In Figure 3: "Lanes 2-7: RNA samples treated with different concentrations of borax." What is the concentration of borax tested in each lane?	**Done.** This figure has been renumbered to **Figure 2** in the revised manuscript. I have added a detailed legend for **Figure 2** that explicitly lists the borax concentration tested in each lane (Lanes 1-7: 0 mM up to300 µM) to ensure full reproducibility.

I trust that the revised manuscript and these detailed responses have adequately addressed all concerns. I am grateful for the opportunity to revise and resubmit my work to *PLOS ONE*.

Sincerely,

Abdulhadi Albaser
